# Antimicrobial, antioxidant, and antitumor activity of epsilon-poly-L-lysine and citral, alone or in combination

**DOI:** 10.3402/fnr.v60.31891

**Published:** 2016-06-15

**Authors:** Ce Shi, Xingchen Zhao, Zonghui Liu, Rizeng Meng, Xiangrong Chen, Na Guo

**Affiliations:** 1 Department of Food Quality and Safety, Jilin University, Changchun, P. R. China; 2 Jilin Entry-Exit Inspection And Quarantine Bureau, Changchun, P. R. China

**Keywords:** ε-PL, citral, synergism, antimicrobial, antioxidant, antitumor, food industries

## Abstract

**Background:**

Food safety is an important worldwide public health concern, and microbial contamination in foods not only leads to food deterioration and shelf life reduction but also results in economic losses and disease.

**Objective:**

The main aim of the present study was to evaluate the effect of epsilon-poly-L-lysine (ε-PL) and citral combination against *Escherichia coli* O157:H7 (*E. coli* O157:H7) strains. The preliminary antioxidant and antitumor activities were also studied.

**Design:**

Synergism is a positive interaction created when two compounds combine and exert an inhibitory effect that is greater than the sum of their individual effects. The synergistic antimicrobial effect of ε-PL and citral was studied using the checkerboard method against *E. coli* O157:H7. The minimal inhibitory concentration, time-kill, and scanning electron microscope assays were used to determine the antimicrobial activity of ε-PL and citral alone or in combination; 2,2-diphenyl-1-picrylhydrazyl-scavenging assay and western blotting were used in antioxidant activity assays; cell viability assay was carried out to finish preliminary antitumor test.

**Results:**

Minimal inhibitory concentrations of ε-PL and citral resisted to the five *E. coli* O157:H7 strains were 2–4 µg/mL and 0.5–1 µg/mL, and the fractional inhibitory concentration indices were 0.25–0.375. The results of time-kill assay revealed that a stronger bactericidal effect in a laboratory medium might be exerted in the combination against *E. coli* O157:H7 than that in a food model. The compounds alone or in combination exhibited a potential 2,2-diphenyl-1-picrylhydrazyl radical–scavenging activity, and the expression of superoxide dismutase 1 and glutathione peroxidase 1 protein increased. The preliminary antitumor activity effect of the combination was better than ε-PL or citral alone.

**Conclusions:**

These findings indicated that the combination of ε-PL and citral could not only be used as a promising naturally sourced food preservative but also be used in the pharmaceutical industry.

Food safety is an important worldwide public health concern, and microbial contamination is a vital factor not only causing food deterioration and shelf life reduction but also resulting in economic losses and disease (1, 2), including intestinal disorders, vomiting, and diarrhoea (3). Outbreaks of food-borne pathogens, such as *Escherichia coli* O157:H7 (*E. coli* O157:H7), continue to draw public attention to food safety. *E. coli* O157:H7 strains can cause watery diarrhea, hemorrhagic colitis, hemorrhagic uremic syndrome, and thrombotic thrombocytopenic purpura (2). In recent years, extensive data from the epidemiologic survey disclosed that the presence of *E. coli* O157:H7 in external environments, such as excrements, sewages, foods, and soils, for growing vegetables has been widely documented in China and other countries of the world (4). Therefore, preservation of food materials from degradation, mainly by microorganism activity, during production, storage, and marketing is an important issue in food industries. Nowadays, although chemical synthetic preservatives show good antimicrobial activity, there is a growing recognition that the continuous use of chemical synthetic preservatives in food industry may result in various hazards to human health (5), and they are restricted due to their carcinogenicity (6). Thus, there has been increasing interest and priority in finding natural, effective, and safe food preservatives, since they can improve the safety of food products for decades (7) and protect the human body by retarding the progress of many chronic diseases, including cancer (8).

Epsilon-poly-L-lysine (ε-PL) produced by *Streptomyces* or *Kitasatospora* is a homopolymer of l-lysine with a polymerization degree of 25–35 connected via the ε-amino and α-carboxyl groups of l-lysine (9), and it is also biodegradable, edible, water soluble, thermally stable, and nontoxic to humans (10). ε-PL is a promising natural antimicrobial that is widely used to preserve packaged food in certain countries for its broad antimicrobial activity against Gram-negative and Gram-positive bacteria, yeasts, and molds (11). Based on its strong antibacterial activity and low toxicity, ε-PL has been used in Japan, the United States, and Korea with a growing demand (10). Hence, this biopolymer is very desirable as a preservative in the food industry.

Citral (3,7-dimethyl-2,6-octadienal) is a mixture of two isomers, geranial and neral, which are acyclic α, β-unsaturated monoterpene aldehydes naturally occurring in many essential oils from citrus fruits or other herbs or spices (12). The antimicrobial action exerted by citral against bacteria and fungi in different conditions has already been demonstrated (13). Due to its prevalence in many industries, citral has long been accepted by Western regulatory bodies in the US and Europe. As a result, it has been afforded the status of ‘generally recognized as safe’ (GRAS) (14).

To the best of our knowledge, as far as the literature is concerned, ε-PL and citral alone resisted to *E. coli* O157:H7 strains have been studied previously, but not in combination. The increasing bacterial resistance to antibiotics represents the main factor justifying the need to find and develop new antimicrobial agents. Antimicrobial combination therapy may be used to extend spectrum coverage, prevent the emergence of resistant mutants, and gain synergy between antimicrobials (15). Moreover, there were no reports on the antioxidant and antitumor activity of ε-PL and citral alone or in combination. Therefore, in this research, our primary objective was to evaluate the antimicrobial activity of ε-PL and citral alone or in combination. Antioxidant and antitumor activities were also studied.

## Materials and methods

### Chemical reagents

ε-PL was purchased from Ruitaibio (Beijing, China) nd citral was purchased from Dr. Ehrenstorfer GmbH (Augsburg, Germany). Lysogeny broth (LB) and Mueller–Hinton (MH) broth were obtained from Qingdao Hope Bio-Technogy Co., Ltd (Qingdao, China). Dulbecco's modified Eagle's medium (DMEM) obtained from Corning (New York, USA) and fetal bovine serum (FBS) obtained from Invitrogen-Gibco (New York, USA) were used for HepG2 cells and L-02 cells culture. Anti-superoxide dismutase 1 (SOD1) and anti-glutathione peroxidase 1 (GPx1) were purchased from Abcam (Cambridge, UK). The 2,2-diphenyl-1-picrylhydrazyl (DPPH) were purchased from Sigma-Aldrich (Saint Louis, USA). The carrots were purchased from the local supermarket (Changchun, China).

### Bacterial strains, cells, and culture conditions

Five *E. coli* O157:H7 strains (944, 932, 380, H1730, and ATCC 35150) used in this study were obtained from Jilin Entry-Exit Inspection and Quarantine Bureau, and maintained at 4°C on slants of LB agar. After two transfers of *E. coli* O157:H7 strains in LB at 37°C for 24 h, the activated cultures of *E. coli* O157:H7 strains were inoculated into LB and incubated at 37°C for 12–18 h. The L-02 and HepG2 cells, purchased from Type Culture Collection of the Chinese Academy of Sciences (Shanghai, China), were cultured in DMEM containing 10% FBS at 37°C in a 5% CO_2_ atmosphere.

### Determination of MIC values of the antimicrobial agents

The minimal inhibitory concentration (MIC) values of ε-PL and citral resisted to the five *E. coli* O157:H7 strains were determined by standard broth microdilution susceptibility testing method according to previous studies (16) and described by the Clinical and Laboratory Standards Institute guidelines (17). The MIC values were defined as the lowest concentration of antimicrobial that inhibited the growth of microorganism by visual reading.

### Checkerboard assay

Mechanistic interactions between compounds are usually measured with the broth dilution checkerboard assay. A checkerboard assay, performed in a checkerboard configuration in a 96-well microtiter plate by broth microdilution, was determined for the interactive inhibition between ε-PL and citral of different classes, and the assay was in conformance with established procedures (18). The concentration of ε-PL and citral used in this assay ranged from 0.25 to 8 µg/mL and 0.125 to 8 µg/mL, respectively. The serial twofold dilutions of compounds against the organism were to be tested. The inoculums were adjusted to final concentrations of 5×10^5^ CFU/mL for each well, and the plate was incubated at 37°C for 24 h. The CLSI guidelines were used to ensure the accurate microbiological assay. In order to evaluate the antibacterial effects of each combination, the fractional inhibitory concentration indices (FICIs) were calculated as the ratio of the MIC of agents A and B in combination with the MIC of agent A (or B) alone. The FICIs were calculated as follows:

$$\text{FICIs}=\text{FIC}{\text{I}}_{\text{A}}+\text{FIC}{\text{I}}_{\text{B}}=({\text{C}}_{\text{A}}^{\text{COMB}}/\text{MI}{\text{C}}_{\text{A}})+({\text{C}}_{\text{B}}^{\text{COMB}}/\text{MI}{\text{C}}_{\text{B}})$$

where MIC_A_ and MIC_B_ are defined as the MIC of agents A and B acting alone, and ${\text{C}}_{\text{A}}^{\text{COMB}}$ and ${\text{C}}_{\text{B}}^{\text{COMB}}$ are the MICs of agents A and B when in combination. The sum of FICIs of two compounds in the combination was calculated as follows: FIC_A_+FIC_B_=FICIs. FICI ≤0.5, synergy; 0.5<FICI≤4, indifference; FICI>4, antagonism (19). The results were expressed in log of CFU/mL.

### Time-kill assay in LB and carrot juice

The objective of this study was to investigate the effect of ε-PL, alone or in combination with citral, on *E. coli* O157:H7 ATCC 35150 in laboratory medium and in carrot juice as a food model to develop a better understanding of the antimicrobial activity. The time-kill assay was performed in four test tubes containing an initial inoculum of 10^6^ CFU/mL in LB and carrot juice with a single or a combination of the compounds with modification according to previous methods. The tube contained bacteria only served as a control (20). The bacterial counts were determined after predetermined time points (0, 3, 6, 9, 12, and 24 h) of incubation by spreading appropriate dilutions on LB agar plates to allow for growth. The plates were incubated at 37°C overnight, and the number of viable cells in each tube was estimated after counting the bacterial colonies on the plates and by multiplying with an appropriate dilution factor. In these steps, all the compounds were performed at ½×MIC. The results from all experiments were conducted in triplicate, and mean values were taken. A bactericidal effect was defined as a ≥3 log_10_ CFU/mL decrease after 24 h of incubation, compared to the density of the initial inoculum. Synergism was defined as a decrease in the colony count of ≥2 log_10_ CFU/mL, with the combination compared to the count obtained with the most active single drug (21).

### Scanning electron microscopy assay

Morphological changes in *E. coli* O157:H7 (ATCC 35150) were observed using scanning electron microscopy (SEM) after treatment with ε-PL and citral alone or in combination. The 1×10^6^ CFU/mL of bacteria was allowed to adhere to polylysine-coated coverslips for about 12 h and treatment with the drugs at ½×MIC alone or in combination for 5–6 h. The cells were washed in PBS after incubation and fixed for 30 min at 4°C with 2.5% glutaraldehyde. The samples were dehydrated in ethanol, freeze-dried with a vacuum freeze drier (Hitachi ES-2030), coated with an ion sputtering apparatus (Hitachi E-1010), and observed through SEM (Hitachi S-3400N). The bacterial cells that were not exposed to the compounds were similarly processed and used as control (22).

### Radical-scavenging activity

This assay was carried out following the same method as reported elsewhere (23) and evaluated by using an ELX800 microplate reader (Bio-Tek, USA). Vitamin C was used as a reference material. All tests were performed in triplicate. Inhibition-free radical DPPH in percent (*I*%) was calculated in the following way:

$$I\%=((A_{\text{DPPH}}-A_{\text{S}})/A_{\text{DPPH}})\times 100$$

where *A*_S_ is the absorbance of the solution containing the sample at 517 nm, and *A*_DPPH_ is the absorbance of the DPPH solution. The results were expressed in EC_50_ values (sample concentration providing 50% of antioxidant activity), which was calculated from the linear regression algorithm of the graph plotted inhibition percentage. Lower EC_50_ values mean greater antioxidant activity.

### Western blotting for SOD1 and GPx1 protein analyses

The L-02 cells (1×10^6^ cells) were seeded in a 100-mm plate and cultured overnight. Then, the cells were exposed with ε-PL and citral, alone or in combination, for 24 h. No compounds treatment served as a control. Cellular proteins were extracted and quantified by BCA kit (KeyGEN Biotech, China). Western blotting was performed using 60 µg of protein sample and antibodies against SOD1 and GPx1 (abcam, USA; 1:1,000); β-action served as an internal control (Sigma; 1:10,000) (24). Bands were visualized by chemiluminescence detector (DNR, Kiryat Anavim, Israel). To obtain accurate results, protein blots were measured and analysed using Image J software (25).

### Preliminary antitumor test: cell viability assay

Cell viability after ε-PL and citral, alone or in combination, treatment was assessed by using 3-(4,5-dimethylthiazol-2-yl)-2,5-diphenyltetrazolium bromide (MTT) dyereduction assay and optical density determined at 490 nm. All cells were seeded in 96-well plates at 1×10^6^ cells per well and allowed to attach to plates overnight. Cells were incubated with the drugs at various concentrations for 48 h. Drug-free served as control. After the respective incubation period, the cells were treated with MTT (5 mg/mL in PBS) for 4 h at 37°C. Then, 150 µL DMSO per well was added to dissolve the formazan. The viable cell number is directly proportional to the production of formazan, which was read as the absorbance value at 490 nm using a microplate reader (Bio-Tek, USA) (26).

### Statistical analysis

All measurements were made in triplicate and each experiment was performed on three separate occasions; the results are expressed as the average of the three parallel assays. A Student's *t*-test was computed to determine the statistical significance of the results. Differences were judged to be statistically significant when *p*<0.05. Statistical analysis was conducted using SPSS 11.5 statistical software.

## Results

### The interaction of antimicrobials against *E. coli* O157:H7 strains

The *in vitro* activities of ε-PL and citral alone or in combination against *E. coli* O157:H7 strains were summarized in Table 1. In this assay, the MICs of ε-PL against *E. coli* O157:H7 strains ranged from 2 to 4 µg/mL, showing ε-PL had a significant antibacterial activity at a very low dosage and the MIC values of citral ranged from 0.5 to 1 µg/mL. Furthermore, the combination of ε-PL and citral further lowered the MICs of individual agent. In checkerboard assay, FICI was used to analyze the interaction of the combinations. The interactions between ε-PL and citral against the five *E. coli* O157:H7 strains were synergistic (FIC, ≤0.5) with FICI values ranging from 0.25 to 0.375. No antagonism (FIC, >4) was observed.

**Table 1:** Synergistic effects of ε-PL and citral alone or in combination against *E. coli* O157:H7 strains

		MICs of compounds (µg/mL)			
				
*E. coli* O157:H7 strains	Agent	Alone	Combination	FICI	Outcome
944	ε-PL	2	0.5	0.375	Synergism
	Citral	1	0.125		
932	ε-PL	4	0.5	0.375	Synergism
	Citral	1	0.25		
380	ε-PL	2	0.25	0.375	Synergism
	Citral	0.5	0.125		
H1730	ε-PL	2	0.25	0.25	Synergism
	Citral	1	0.125		
ATCC 35150	ε-PL	2	0.25	0.375	Synergism
	Citral	1	0.25		

### Time-kill assays

**Fig. 1:**
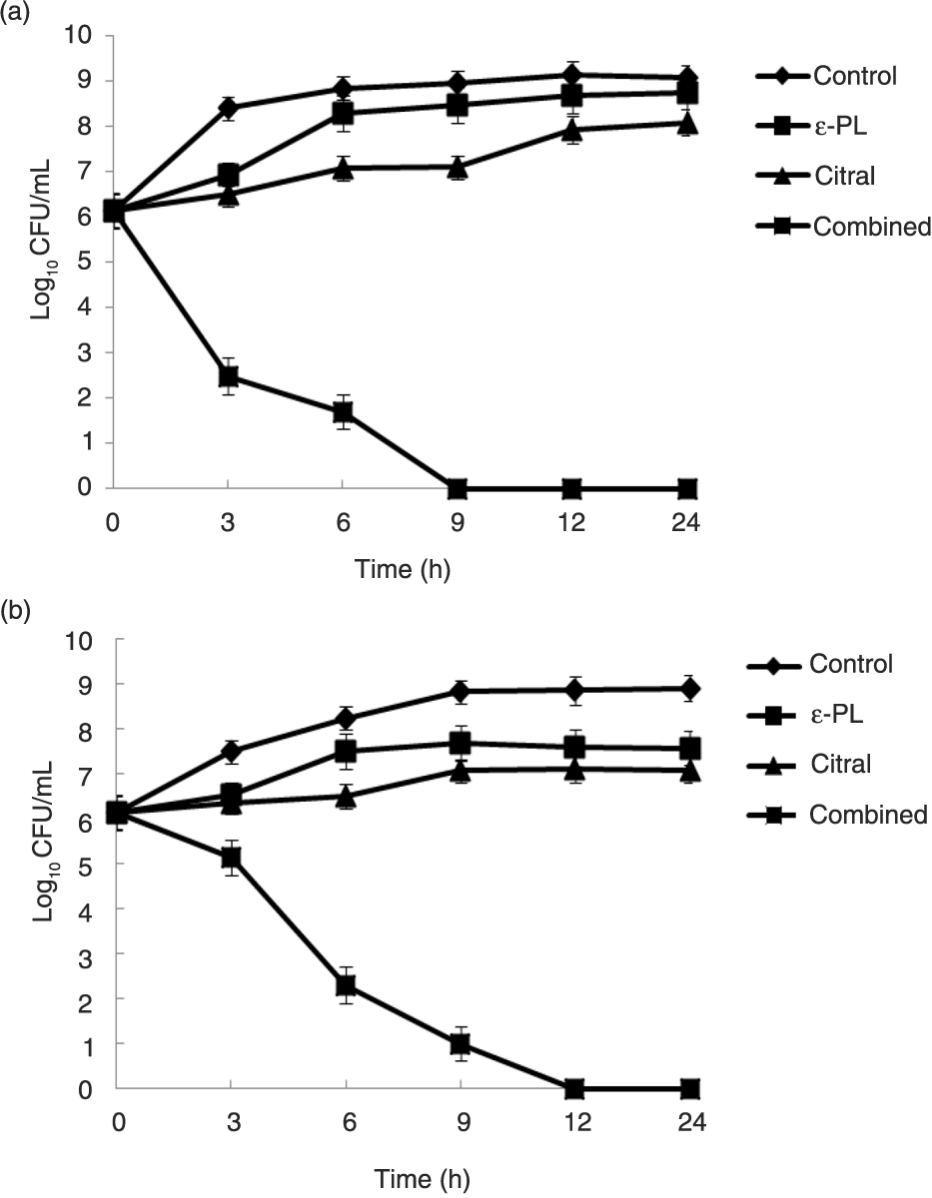
Time-kill curves for ε-PL and citral alone or in combination against *E. coli* O157:H7 ATCC 35150 in laboratory medium (a) and carrot juice (b). The strains were exposed to *in vivo* concentrations of 1 µg/mL ε-PL, 0.5 µg/mL citral, and 1 µg/mL ε-PL+0.5 µg/mL citral at a starting inoculum of 10^6^ CFU/mL. Values are the means of three independent experiments with the standard deviation indicated by vertical bars.

The bacterial effect of ε-PL combined with citral against *E. coli* O157:H7 ATCC 35150 was confirmed by time-kill curve experiments. Time-kill assays for the synergistic combinations on *E. coli* O157:H7 ATCC 35150 in LB and carrot juice were shown in Fig. 1. The time-kill assays were conducted to determine the rates of killing of test bacteria when exposed to ε-PL and citral alone or in combination. Figure 1a showed the results of time-kill assay in laboratory medium, which expressed the effect of ε-PL and citral alone or in combination on the growth of *E. coli* O157:H7 ATCC 35150 in LB. If ε-PL or citral was used alone, there was no significant growth inhibition. However, if ε-PL and citral were used in combination, the bacteriostatic activity dramatically increased. Given an initial inoculum of 10^6^ CFU/mL, the combination therapy of ε-PL and citral yielded an 8.1 log_10_ CFU/mL decrease compared with citral alone at 24 h for *E. coli* O157:H7 ATCC 35150. Fig. 1b showed the effect of ε-PL and citral alone or in combination on the growth of *E. coli* O157:H7 ATCC 35150 in carrot juice. Similarly, if ε-PL or citral was used alone, there was almost no significant growth inhibition. But a strong bactericidal effect was exerted in compounds combination. For example, the combination resulted in a 7.09 log_10_ CFU/mL decrease compared with citral alone at 24 h.

### SEM observation

**Fig. 2:**
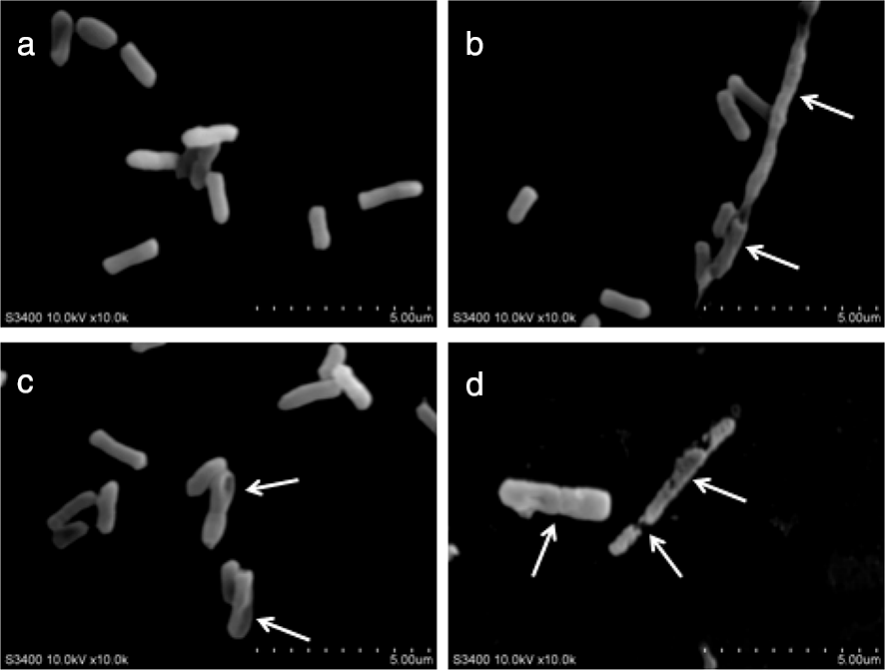
SEM of *E. coli* O157:H7 ATCC 35150 exposed to ε-PL and citral alone or in combination. (a) Control cells (no treated), (b) cells treated with ε-PL (1 µg/mL), (c) cells treated with citral (0.5 µg/mL), and (d) cells treated with ε-PL and citral (1 µg/mL+0.5 µg/mL).

SEM analysis revealed notable morphological changes due to the exposure to ε-PL and citral alone or in combination (Fig. 2). The control cells, without ε-PL or citral treatment, displayed normal bacillary morphology with a smooth, regular surface (Fig. 2a). The cells treated with ε-PL showed sunk and distorted forms with strongly rugged surfaces (Fig. 2b) that differed from the control cells. After treatment with citral, some holes were found on the surface of *E. coli* O157:H7 (944), and a distorted, irregular shape was observed (Fig. 2c). Furthermore, the effects of ε-PL in combination with citral on *E. coli* O157:H7 were the most severe, cells with a normal shape were difficult to find, and the majority of cells had been changed. Cell wall deterioration and a high degree of cell lysis were especially observed (Fig. 2d). These findings demonstrate that if ε-PL combined with citral, cells external modifications were more severe than ε-PL or citral alone.

### Free radical–scavenging activity

The DPPH free radical is a stable free radical, which has been widely accepted as a tool for estimating the free radical–scavenging activity of antioxidants. The classification of additive, synergistic, or antagonistic effects was performed as follows. Additive: EC_50_ theoretical and experimental values reveal differences lower than 10%; synergistic: EC_50_ experimental values are more than 10% lower than theoretical values; antagonistic: EC_50_ experimental values are more than 10% higher than theoretical values. The limit of 10% was chosen taking into account the coefficients of variation obtained in the replications of each antioxidant activity assay. Theoretical values for antioxidant activity of the mixtures were calculated as weighted mean experimental EC_50_ values of the individual samples, considering additive contributions of individual species in each percentage (23). In this study, ε-PL (50%)+citral (50%) EC_50_=EC_50_ ε-PL×0.5+EC_50_ citral×0.5. According to the results obtained, the EC_50_ of ε-PL and citral alone was 17.63 µg/mL and 38.01 µg/mL after calculation, respectively. However, compared to the compounds alone, the DPPH radical–scavenging activity of the combination was found higher (EC_50_=6.02 µg/mL). After calculation, the theoretical value of the combination was 22.526 µg/mL. It represented that synergistic effects between ε-PL (50%) and citral (50%) was observed. The results indicated that the compounds alone or in combination exhibited a potential DPPH radical–scavenging activity.

### The compounds alone or in combination increased SOD1 and GPx1 protein expression in L-02 cells

**Fig. 3:**
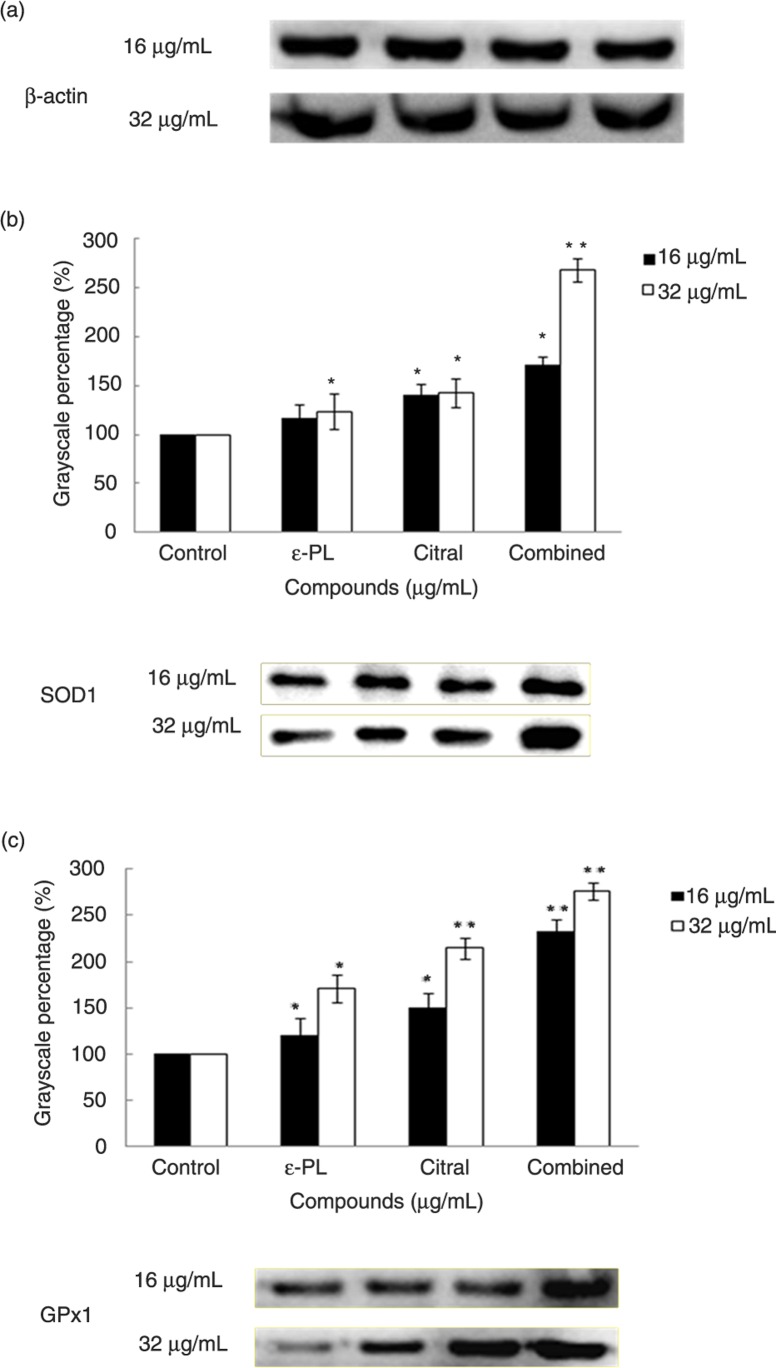
Western blot analysis of SOD1 (a) and GPx1 (b) production in L-02 cells treated with different concentrations of ε-PL and citral alone or in combination. To obtain accurate results, protein blots were measured using the Image-J software. The values show the means±SD for three independent experiments (*p<0.05, **p<0.01).

L-02 cells were treated with the compounds alone or in combination at concentrations of 16 µg/mL and 32 µg/mL for 24 h. Protein expression of SOD1 and GPx1 in L-02 cells was analyzed with western blotting. As shown in Fig. 3, compared to the control, a concentration-dependent upregulation of SOD1 and GPx1 protein expression by the compounds alone or in combination could be seen. And actually, the gray-scale values for the control groups were the same for the expression of SOD1 and GPx1 during 16 µg/mL and 32 µg/mL of ε-PL and citral, alone or in combination. Therefore, the control groups of 16 µg/mL and 32 µg/mL treatment served as 100%. The gray-scale levels of SOD1 and GPx1 visibly increased compared to the control. In addition, through treatment with the combination of ε-PL and citral, the expression of SOD1 and GPx1 protein was upregulated compared to ε-PL or citral alone in a dose-dependent manner.

### Cell viability assay

**Fig. 4:**
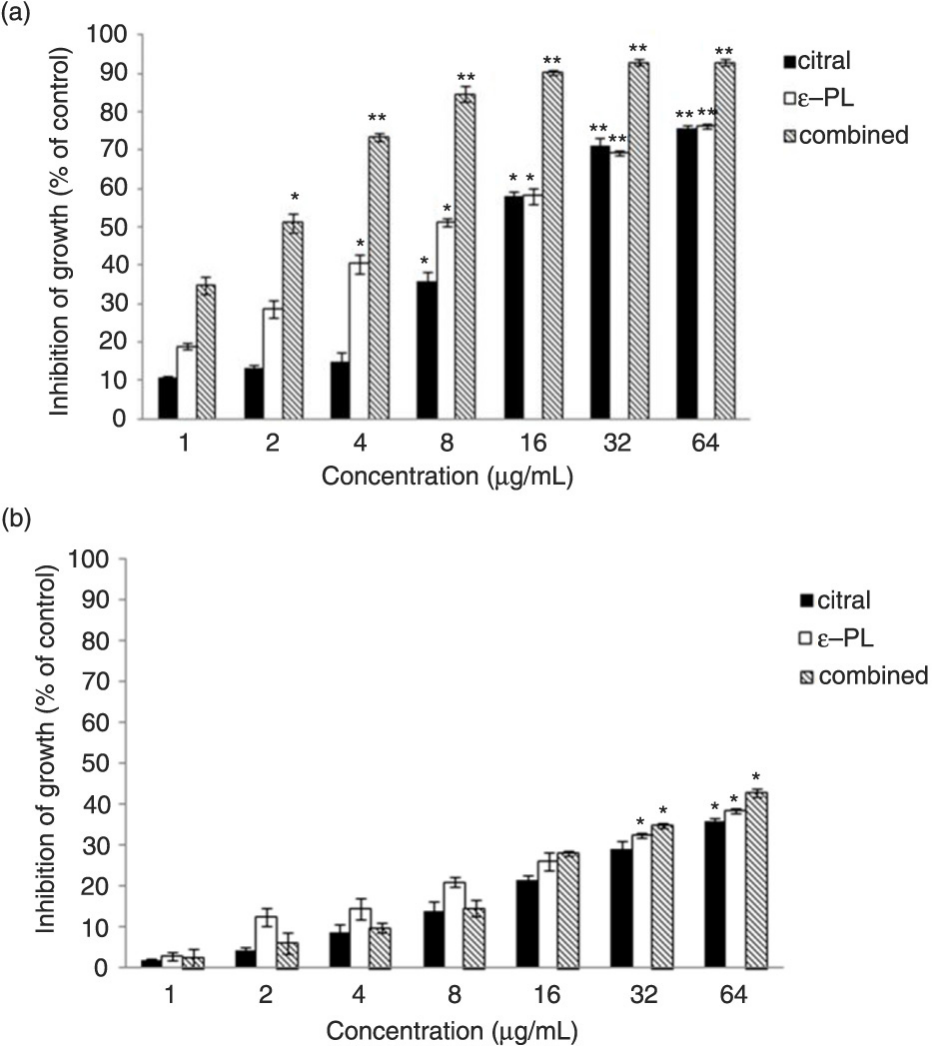
Cytotoxic activity of ε-PL and citral, alone or in combination, treatment for 48 h on (a) HepG2 cells line and (b) L-02 cells. Data were expressed as mean±SD of three different experiments performed in triplicate. *Error bars* indicate the standard deviation (*p<0.05, **p<0.01).

To investigate the tumoricidal activity of citral and ε-PL, the effects on the viability of cancer cell line (HepG2 cells) and a normal cell line (L-02 cells) were evaluated. As shown in Fig. 4, for HepG2 cells, the 50% inhibiting concentration (IC_50_) for 24 h and 48 h of ε-PL treatment was 13.49 µg/mL and 8.664 µg/mL, respectively; citral treatment was 30.129 µg/mL and 14.67 µg/mL, respectively; and the combination treatment was 3.331 µg/mL and 1.691 µg/mL, respectively. In comparison with the cytotoxic effects on HepG2 cells and L-02 cells, it indicated that ε-PL and citral, alone or in combination, exhibited less cytotoxicity to normal L-02 cells and inhibited HepG2 cells in a dose-dependent manner. Especially important, the preliminary antitumor activity effect of the combination was better than ε-PL or citral alone.

## Discussion

Our study indicated that the combination of ε-PL and citral was more effective as antimicrobials than each used alone. The synergy of ε-PL and citral against *E. coli* O157:H7 strains has not been reported before. From our results, FIC indices assays suggested a synergistic effect of the combined application of ε-PL and citral against *E. coli* O157:H7 in carrot juice and TSB. *In vitro* time-kill studies, one of the most commonly used experimental models to assess synergetic antibacterial activity, efficiently characterize the rate, extent, and timing of bacterial killing and regrowth (27). From the data of SEM assay, nisin and CA not only caused morphological changes of *E. coli* O157:H7 individually but also showed synergistic interaction. From the results, we concluded that ε-PL and citral alone or in combination could inhibit the growth of *E. coli* O157:H7 strains. Zhou et al. (28) indicated that the MIC value of ε-PL for *E. coli* was 8 µg/mL, and Shima et al. (9) and Hiraki (29) reported that MICs of ε-PL against *E. coli* K-12, *E. coli* F-2, and *E. coli* B were 1 µg/mL, 2 µg/mL, and 1 µg/mL. The results of our study were within this range. However, the details of the modes of action and the molecular mechanism that causes the death of the target cell are not fully understood, thus delaying the emergence of the combination of the compounds as a new class of antibiotics. Previous studies have inferred that the antimicrobial activity of the compounds might be associated with cell membrane damage (30). The cell wall is the first barrier for bacteria to the external environment, which plays an important part in maintaining the morphology and protecting the cell from the harmful substances. Hence, it was very important to investigate the biological effect of ε-PL and citral on the surface of bacteria. At the same time, the underlying mechanism of the compounds alone or in combination against *E. coli* should be investigated further.

A previous study demonstrated that adding antioxidants effectively slows down the oxidation of food or even deters corruption, and antioxidants that retard the oxidation process may additionally exhibit antimicrobial activity. Antioxidants can protect the human body from the progress of many diseases and prevent, stop, or reduce oxidative damage by scavenging free radicals and diminishing oxidative stress (31). Free radicals have been implicated in over a hundred disease conditions in humans, including hemorrhagic shock, atherosclerosis, ischemia, reperfusion injury to many organs, tumor promotion, and carcinogenesis (32). Thus, the free radical–scavenging capacity of the compounds against common free radicals (DPPH) *in vitro* was further determined. DPPH is a free radical compound that has been widely used to determine the free radical–scavenging capacity of various samples because of its stability (in radical form), simplicity, and fast assay. Reactive oxygen species (ROS), such as superoxide anions, hydroxyl radicals, and hydrogen peroxide, are free radicals produced as byproducts of redox reactions. When the physiological balance between ROS production and antioxidant defences is lost during exposure to stressful stimuli, oxidative stress subsequently results in damage to nucleic acids, proteins, and lipids (33). Within cells, one of the means to control excessive ROS formation is their degradation by antioxidant enzymes. In the antioxidant system, SOD1 and GPx1, the major enzymes responsible for the inactivation of superoxide and hydrogen peroxide, respectively, enable the scavenging of free radicals (24). In this study, we reported, for the first time, that ε-PL and citral alone or in combination upregulate the protein expression of SOD1 and GPx1 in L-02 cells.

In addition, to our knowledge, liver cancer is one kind of high-grade malignant tumor. Current estimates indicate that liver cancer is the sixth most common cancer worldwide and the third leading cause of cancer-related deaths. At present, curative resection is still the main treatment of liver cancer patients. However, the success of this approach is limited by the tumor size and the function left as well as the metabolism of the liver. Furthermore, the resection has a high relapse rate and is also unsuitable to the late-stage patients (34). Thus, finding a medicine for tumor cells and understanding the underlying mechanisms of its anticancer properties are of great significance.

## Conclusions

In conclusion, the combination of ε-PL and citral displayed good synergistic antibacterial activities against *E. coli* O157:H7 strains, which increased the effectiveness of antibacterial compared alone. In this study, the results revealed that a stronger bactericidal effect in a laboratory medium might be exerted in the combination of ε-PL and citral against *E. coli* O157:H7 strains than that in a food model. Although the antibacterial mechanism about morphological changes of *E. coli* O157:H7 strains was studied, further research of the mechanism should be carried out in the future. In addition, the compounds showed better antioxidant and antitumor activity. These findings indicate that the combination of ε-PL and citral could not only be used as a promising naturally sourced food preservative but alsobe used in the pharmaceutical industry.
